# Nephropleural Fistula Effectively Managed with Serial Thoracentesis: A Case Report

**DOI:** 10.1089/cren.2016.0102

**Published:** 2016-11-01

**Authors:** Aaron D. Baugh, Eslam Youssef, Syed Shafae Hasan, Nauman Saleem Siddiqui, Haitham Elsamoloty, Khaled Shahrour, Toseef Javaid

**Affiliations:** ^1^ Department of Internal Medicine, University of Toledo College of Medicine, Toledo, Ohio.; ^2^ Department of Radiology, University of Toledo College of Medicine, Toledo, Ohio.; ^3^ Department of Urology, University of Toledo College of Medicine, Toledo, Ohio.

**Keywords:** complications, nephrolithotomy, nephropleural fistula, tube thoracotomy, urinothorax

## Abstract

Nephropleural fistulae are rare but serious thoracic complications of percutaneous nephrolithotomy (PCNL). Herein, we present the management of a 54-year-old female with a delayed presentation of nephropleural fistula. The role of serial thoracentesis as a safe, less invasive, less painful alternative to tube thoracostomy is highlighted. In select cases, this may represent an attractive management strategy for nephropleural fistula after PCNL.

## Introduction

Nephropleural fistula describes an abnormal communication between the pleural space and renal collecting system. It is a rare complication of percutaneous nephrolithotomy (PCNL). It subsequently can lead to respiratory distress because of accumulation of large amounts of urine in the chest cavity. Traditionally, it is managed with simultaneous urinary diversion and tube thoracostomy. We present a case of nephropleural fistula effectively managed with serial thoracentesis.

## Case Report

We describe a case of a 54-year-old Caucasian female who presented with abdominal pain from her recurrent nephrolithiasis. She had a right 2.1 cm partial staghorn calculus and 1 cm left lower pole calculus. She underwent right PCNL. Under fluoroscopic guidance, the right upper calix was accessed through an intercostal approach between the 11th and 12th ribs and had a right percutaneous nephrostomy tube placed at the end of the procedure to maintain percutaneous renal access for possible second look nephroscopy. Ureteral stent was not placed as there was no indication for stent placement especially that nephrostomy tube was placed. There were no immediate postoperative complications, and a routine postoperative chest radiograph was unremarkable. The next day, the patient complained of dyspnea and worsening pain. Routine postoperative computed tomography (CT) scan showed two small residual stones in right ureter in addition to the previously known left renal stone. A chest X-ray done on postoperative day (POD) 2 was unremarkable except for atelectasis. The pain management service was consulted. For complete stone removal, she was taken to surgery on POD 3 for a second-look cystoscopy with bilateral ureteroscopy. Residual stones were removed from the right kidney along with the left renal stone. A left ureteral stent was placed at the end of the procedure. No stent was placed in the right ureter as she already had the nephrostomy tube in place, the right ureter was patent at the end of right ureteroscopy with minimal traumatization, there was no suspicion for any pleural injury at that time, and we did not want the patient to be bothered by having bilateral stents. The patient's pain improved and the patient was discharged from the hospital on POD 4 after removal of her right percutaneous nephrostomy tube. She was discharged with the indwelling left ureteral stent in place with removal planned in 1 week in clinic.

**FIG. 1.**
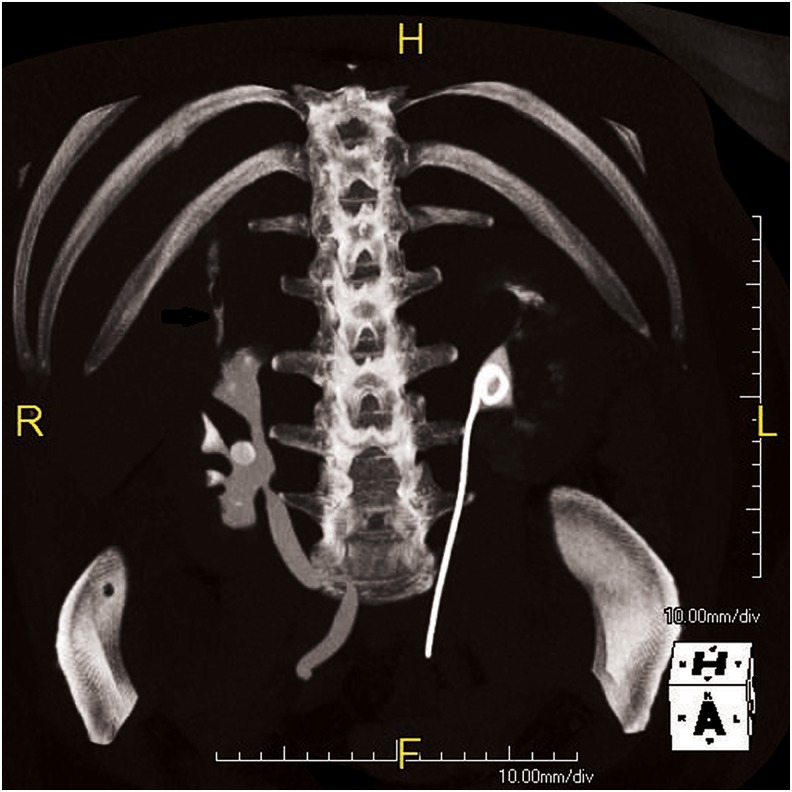
Axial cut of CT abdomen/pelvis with *arrow* showing fistula. CT, computed tomography.

**FIG. 2.**
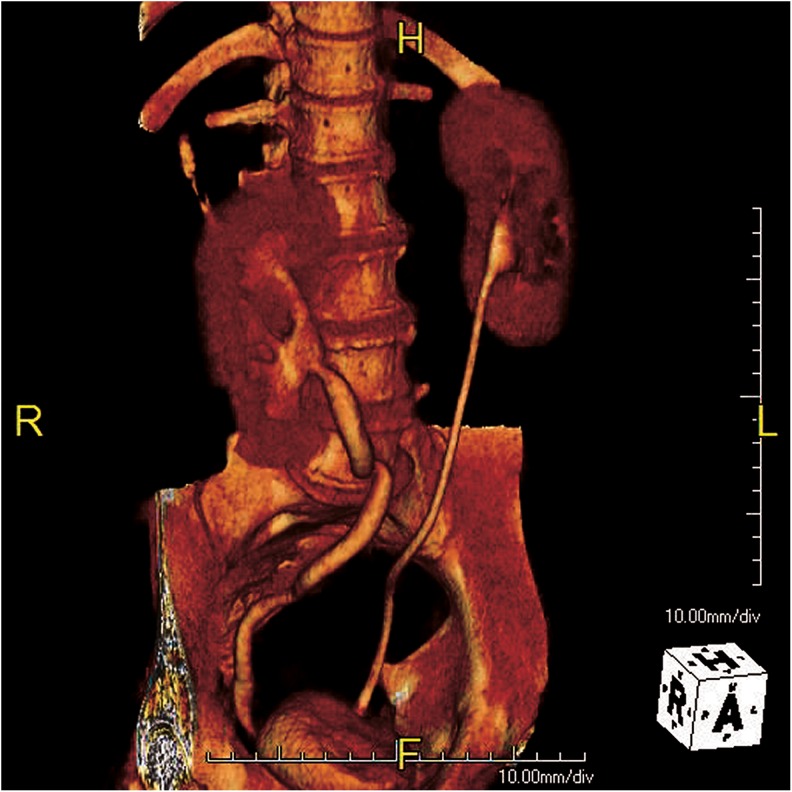
3D reconstruction of fistulous tract.

**FIG. 3.**
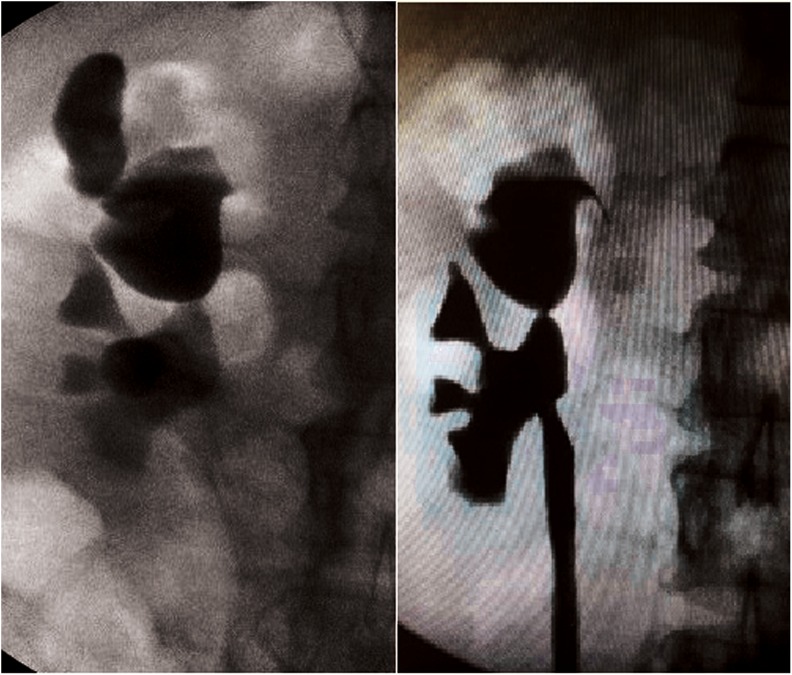
Retrograde pyelogram from before and after fistula healing. “Before” image shows contrast spillage.

The patient was readmitted a day later for respiratory distress that required supplemental oxygen through a nonrebreather face mask. A CT scan of the chest, abdomen, and pelvis with contrast showed a large right pleural effusion with a fistulous tract between the right kidney and right pleura (Figs. 1 and 2). The patient expressed serious reservations about pain control given her difficulties in the previous admission, and preferred less invasive options when management strategies, including tube thoracostomy, were discussed. Emergent therapeutic thoracentesis was performed instead and 2000 cc of pleural fluid was drained. The patient's respiratory distress significantly improved after thoracentesis. Pleural fluid analysis showed low fluid pH, and a fluid-to-serum creatinine ratio of around 4.5, both consistent with urinothorax.. The patient had another thoracentesis on POD 6 from her initial surgery and 700 cc pleural fluid was again drained. On the following day, the patient returned to the operating room and a 6F, 26 cm Double-J ureteral stent was placed in the right ureter along with 16F urethral catheter to prevent urine reflux with voiding. The patient thereafter demonstrated gradual improvement in her symptoms without reaccumulation of fluid in the pleural cavity and was subsequently discharged home in stable condition. Her Foley catheter was removed 2 weeks later. Retrograde pyelography at 1 month follow-up showed complete resolution of her nephropleural fistula and the stent was removed at that time (Fig. 3). At about 6 months from the said visit, the patient has not subsequently had respiratory complaints.

## Discussion

Nephropleural fistula is an abnormal communication between the pleural cavity and renal collecting system because of direct injury to the parietal pleura. Some case reports note detection of a fistula soon after nephrostomy tube removal or displacement, presumably because of urine following the path of least resistance.^1^ This was a more delayed presentation.

In a large retrospective review, the reported incidence of nephropleural fistula in PCNL was 0.87%.^2^ Nine cases of nephropleural fistula after PCNL have been well described in the literature. However, it is probably underreported, and with the increasing number of percutaneous urologic procedures, its incidence is expected to rise. Major risk factors for nephropleural fistula include younger age, low body mass index (BMI) because of lack of perirenal fat, and intercostal approaches above the 11th rib.^3^ Neither of these risk factors was present in this case as her intercostal access was below the 11th rib. At variance with the theoretically higher left-sided risk that may be predicted anatomically, the present case saw a right-sided occurrence, in line with Sharma et al. reported increased incidence of pleural injury on right-sided procedures.^3^

Where they required intervention, reported cases in the literature were managed with simultaneous tube thoracostomy and diversion from the urinary tract. Based on patient preference, our patient was treated with serial thoracentesis and urgent ureteral stenting. Contrasting the two procedures, chest tube placement requires longer hospital stay, is less well tolerated from a pain control perspective, and carries a risk of significant intrathoracic complications especially if done by physicians in training.^4^ We propose that in selected cases of uncomplicated pleural effusion because of nephropleural fistula, serial thoracentesis is a safe, effective management strategy when undertaken in combination with urinary tract diversion.

Any patient who presents with shortness of breath and pleural effusion, post-PCNL, should raise suspicion of nephropleural fistula. Currently, immediate postoperative X-rays are recommended to screen for urinothorax and immediate postoperative intrathoracic complications, but these tests can miss such injuries as in our case, most likely because of the presence of the nephrostomy tube covering the injury site and diverting the urine.

## Conclusion

Iatrogenic nephropleural fistula is a rare but potentially life-threatening complication of PCNL. It is more likely to occur with right-sided procedures, when using a supracostal approach especially above the 12th rib, or in patients who are young and those with low BMI.^3^ Although chest tube placement is the most frequently used treatment option, thoracentesis is a less invasive and safer option in selected uncomplicated pleural effusions. Treatment should be understood as divided between emergent symptomatic relief and long-term resolution of pathophysiology. As the least morbid efficacious therapy, thoracentesis should be considered first-line urgent treatment for symptomatic fistulae. Urinary stenting or other forms of diversion should be the next consideration for definitive therapy.

## Abbreviations Used

BMI: body mass index
CT: computed tomography
PCNL: percutaneous nephrolithotomy
POD: postoperative day
